# Resection of the primary tumor with or without liver resection reduces the risk of death in patients with liver metastatic gastroenteropancreatic neuroendocrine tumors: a systematic review and meta-analysis

**DOI:** 10.3389/fonc.2025.1693647

**Published:** 2026-01-14

**Authors:** Yue Xiao, Jianli Wang, Zejin Zhao, Luya Wen, Jian Li, Jinlong Liu

**Affiliations:** 1 Department of Hepatobiliary Surgery, The Affiliated Hospital of Chengde Medical University, Chengde, Hebei, China; 2 Hebei Key Laboratory of Panvascular Diseases, Chengde, Hebei, China

**Keywords:** gastroenteropancreatic neuroendocrine tumors, liver metastases, liver resection, meta-analysis, PTR

## Abstract

**Background:**

Gastroenteropancreatic neuroendocrine tumors (GEP-NETs) patients frequently present with liver metastases (LM) at diagnosis. The benefit of primary tumor resection (PTR) in this context remains controversial. This meta-analysis aimed to quantify the effect of PTR on survival in GEP-NETs patients with LM.

**Methods:**

We performed a systematic search of PubMed and Embase for studies that compared survival outcomes in GEP-NETs patients with LM who underwent PTR versus those who did not. Pooled effects are reported as hazard ratios (HR) with 95% confidence intervals (CI). We also conducted sensitivity analyses to evaluate the robustness of the findings.

**Results:**

Of 1525 screened articles, 11 studies met inclusion criteria, comprising 4185 patients who underwent resection. PTR was associated with improved overall survival compared with non-resection (HR = 0.48, 95% CI: 0.43–0.55). Sensitivity analyses, performed by sequentially excluding individual studies, did not materially change the result.

**Conclusion:**

PTR is associated with increased survival among GEP-NETs patients presenting with LM; however, decisions should be individualized based on patient and tumor characteristics.

## 1 Introduction

Gastroenteropancreatic neuroendocrine tumors (GEP-NETs) are malignant tumors that arise from neuroendocrine cells in the gastrointestinal tract and related organs, though they are relatively uncommon (1). Notably, the incidence of GEP-NETs has risen significantly in recent years (2). Despite being generally considered indolent, these tumors pose diagnostic challenges due to their nonspecific clinical symptoms. Consequently, 40-50% of patients present with distant metastases at diagnosis, with the liver being the most frequent site (3). Furthermore, patients with liver metastases (LM) from GEP-NETs typically have a poorer prognosis compared to those without distant metastases (4).

Pharmacotherapy is currently the primary treatment strategy for patients with GEP-NETs with liver metastases (LM). Available pharmacological options include somatostatin analogues (SMA), molecular targeted therapy, and peptide receptor radionuclide therapy (PRRT) (5–7).

Recent studies have explored the benefits of primary tumor resection (PTR), with or without liver resection (LR), in these patients (8–10). The European Society of Medical Oncology (ESMO) guidelines recommend PTR for patients with GEP-NETs and resectable LM, suggesting it may extend overall survival (OS) (11). However, the effectiveness of PTR remains uncertain. The latest European Neuroendocrine Tumor Society (ENETS) guidelines highlight that in small-intestinal NETs with metastatic disease, palliative or prophylactic PTR is controversial and should be tailored based on symptoms, metastatic burden, and multidisciplinary evaluation (12). Additionally, some clinical studies indicate that PTR in patients with advanced gastrointestinal neuroendocrine tumors (GI-NETs) does not significantly improve survival (13, 14). Thus, further research is needed to determine whether PTR, with or without LR, can effectively prolong OS in patients with GEP-NETs with LM.

This systematic review and meta-analysis aim to assess whether PTR, regardless of liver resection, offers a survival benefit for patients with GEP-NETs with LM. The findings will provide valuable insights for developing personalized treatment strategies.

## 2 Method

### 2.1 Retrieval strategy, inclusion and exclusion criteria

The literature search was performed in accordance with the Preferred Reporting Items for Systematic Reviews and Meta-Analyses (PRISMA) guidelines. We conducted an online search of the PubMed and Embase databases for literature published over the past 20 years. The search strategy employed was: (‘liver Metastasis’ OR ‘liver Metastases’ OR ‘liver Metastatic’) AND (‘neuroendocrine tumor’) AND (‘surgery’ OR ‘resection’), with the search period restricted from May 15, 2005, to May 15, 2025. The complete PRISMA-S checklist and detailed search strategies are available in **Supplementary Tables 1**, **2**.

The inclusion criteria for this study are as follows: (1) Studies involving patients with GEP-NETs with LM; (2) Studies from which the hazard ratio (HR) and its 95% confidence interval (CI) could be obtained, either directly or indirectly; (3) Studies that compared overall survival (OS) between the surgical resection (PTR) group and the non-resection group. Strict exclusion criteria were also established. The literature was limited to studies published in English. Meeting reports, guidelines, case reports, and review articles were excluded. Additionally, single-arm studies were not considered. If multiple publications reported on the same study population, only those with the most comprehensive statistical analyses and the highest quality assessment scores were included. A PRISMA flow chart was subsequently constructed to illustrate the inclusion and exclusion process (15). The detailed PICOS criteria are provided in the **Supplementary Materials** (**Supplementary Table 3**).

### 2.2 Data extraction and quality assessment

All abstracts and full texts were independently extracted by two authors, Y.X. and Z.Z. In cases of disagreement, a third author, J.L.L., was consulted for further evaluation. The quality of the included studies was assessed using the Newcastle-Ottawa Scale (NOS) (16). Each study was independently evaluated by Y.X. and Z.Z., with a NOS score of ≥ 6 deemed indicative of high quality.

### 2.3 Outcomes of interest

The primary outcome of this meta-analysis is the evaluation of OS between the PTR group and the non-resection group. A forest plot was generated to compare the HR of these two groups. Additionally, clinical data from the surgical cohort were collected, encompassing the type of surgery performed and the anti-tumor treatment regimens administered.

### 2.4 Statistical analysis

All statistical analyses were conducted in R (version 4.4.1; R Foundation for Statistical Computing, Vienna, Austria). For studies that did not report HR, we estimated HR from Kaplan–Meier curves using the method of Tierney et al. (17). We performed subgroup analysis by radical resection status and tumor location. Statistical significance was defined as *P* = 0.05, and all tests were two-sided. Heterogeneity was assessed with the *I^2^* statistic: *I^2^* < 20% was considered negligible, and a fixed-effect model was applied; *I^2^* between 20% and 50% indicated acceptable heterogeneity, and a random-effects model was used. Finally, funnel plots and Egger’s test were used to evaluate potential publication bias (18).

## 3 Result

### 3.1 Articles selection

**Figure 1:**
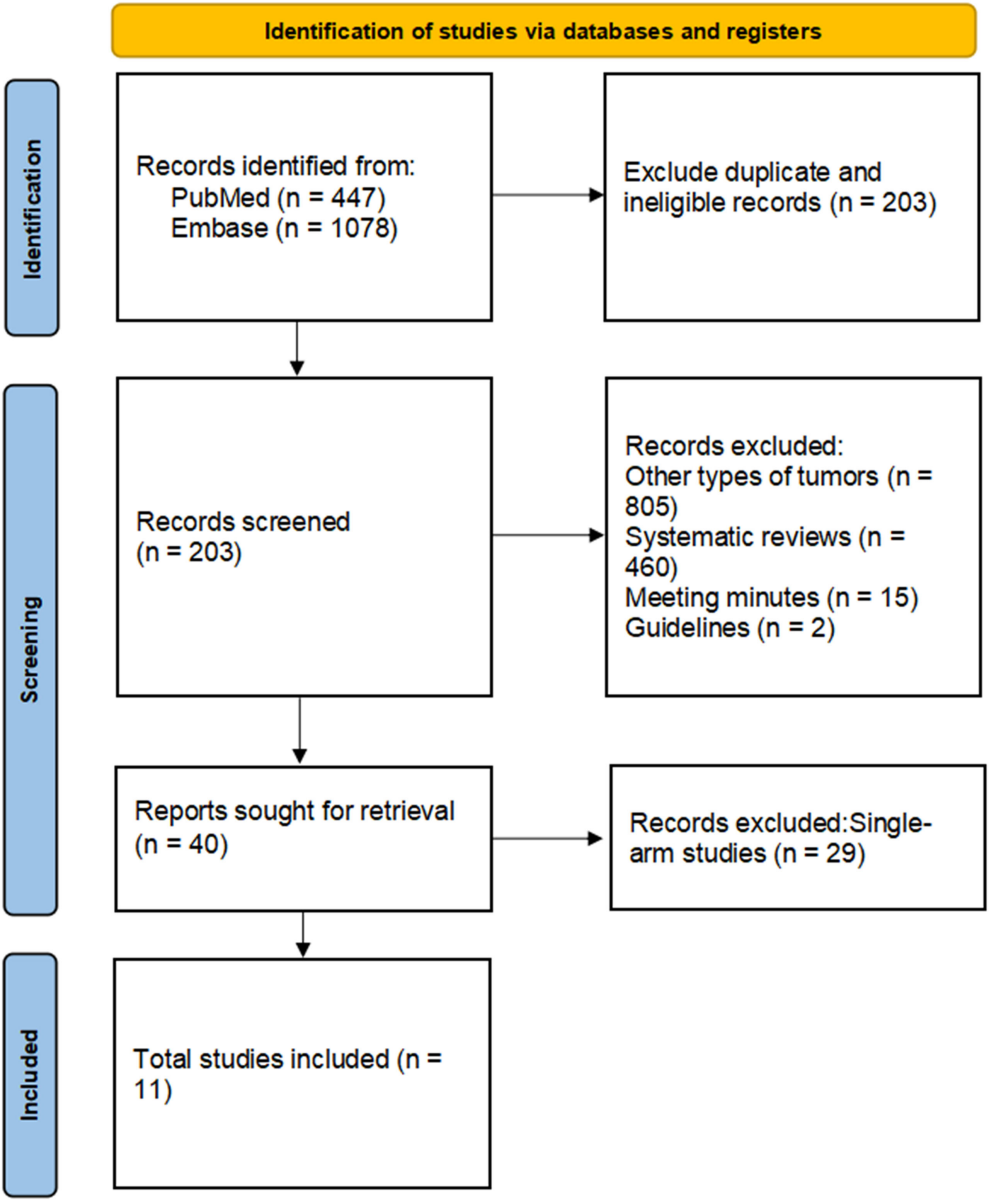
PRISMA diagram of selecting articles.

The preliminary search retrieved 1525 records, of which 203 duplicates were removed. Title and abstract screening excluded 1282 records for reporting other tumor types, being systematic reviews, meeting abstracts, or guidelines. Full-text review of the remaining 40 studies excluded 29 single-arm studies. Thus, 11 studies met the inclusion criteria and were included in the meta-analysis. The study selection flow is shown in **Figure 1**.

### 3 Studies and characteristics

**Table 1** summarizes the final research features included in this study, listing publication year, country, sample size, tumor location, surgical plan, inclusion period, and NOS score. All studies were nonrandomized cohort designs. Five studies (19–23) used data from approved databases, while the remainder were single-center or multi-center investigations (24–29). All studies were rated high quality, and five (23, 25, 27–29) reported additional queues with LR. Surgical cohort sizes ranged from a minimum of 19 patients (24) to a maximum of 1289 patients (21). Three studies (21, 24, 26) did not explicitly report HR, so HR were extracted from Kaplan–Meier curves.

**Table 1:** Baseline characteristics of included studies.

Author	Year	Country	Inclusion period	Study design	No. of resected patients	Tumor location	Burden of disease	Grade (G1/G2/G3)	Surgical protocol	NOS score
Bettini et al. (24)	2009	Italy	1990-2004	Retrospective	19/51	Pancreatic	Only liver	46/NR/5	PTR	7
Partelli et al. (25)	2015	Italy, United Kingdom and Germany	2000-2011	Retrospective	91/166	Pancreatic	Only liver	39/95/32	PTR/PTR + LR	7
Bertani et al. (26)	2017	Italy	1994-2013	Prospective	63/124	Pancreatic	Only liver	6/69/5	PTR	8
Tao et al. (19)	2017	United States	2010-2014	Retrospective	47/191	Pancreatic	Only liver	57/30/10	PTR	7
Lin et al. (27)	2018	China	1998-2016	Retrospective	35/63	Pancreatic	Only liver	9/36/10	PTR/PTR + LR	7
Lewis et al. (28)	2019	United States	2005-2011	Retrospective	392/854	Gastrointestinal	Liver and other metastatic sites	NR	PTR/PTR + LR	7
Zheng et al. (20)	2019	United States	2010-2015	Retrospective	897/1547	Gastroenteropancreatic	Only liver	726/310/352	PTR	7
Gangi et al. (21)	2020	United States	2010-2015	Retrospective	1289/1954	Gastrointestinal	Only liver	992/302/74	PTR	7
Liu et al. (22)	2023	China and United States	2011-2021	Retrospective	55/155	Gastrointestinal	Only liver	21/112/12	PTR	8
Chen et al. (23)	2024	United States	2016-2018	Retrospective	1139/2320	Gastroenteropancreatic	Only liver	NR/576/NR	PTR/PTR + LR	8
Xu et al. (29)	2025	China	1996-2019	Retrospective	93/163	Pancreatic	Only liver	15/106/21	PTR/PTR + LR	7

PTR, primary tumor resection; LR, liver resection.

### 3.3 Surgical selection

Ten studies (19–23, 25–29) described the characteristics of patients who underwent PTR. Five of these studies (19–21, 26, 28) found that younger patients were more likely to receive PTR than older patients. Four studies (20, 22, 23, 28) reported that PTR recipients more often had primary tumors in the small intestine or colorectum. Several studies (20, 22, 23, 28, 29) further noted that patients selected for PTR tended to have moderately to well−differentiated tumors. For pancreatic primaries, PTR was more frequently performed when the tumor was in the pancreatic body or tail (26, 29). When PTR was combined with LR, the liver metastases were typically confined to a single lobe and were small in size (25, 27, 29).

### 3.4 Anti-tumoral medical therapies

Two studies (24, 25) provided detailed data on systemic therapy regimens for patients undergoing surgical resection; one of these studies (24) also reported regimens for the non-resection group, covering 51 patients in total (resection/non-resection = 19/32). In the resection group, somatostatin analogs (SMA; 40.2%) were the most common first-line therapy, followed by chemotherapy (30.4%). After disease progression, chemotherapy (12.5%) and peptide receptor radionuclide therapy (PRRT; 8%) were the preferred options. In the non-resection group, SMA remained the favored treatment (25.8%). Lin et al. (27) recommended systemic therapy for all patients, including octreotide, targeted agents, and/or systemic chemotherapy.

### 3.5 Meta-analysis of overall survival

**Figure 2:**
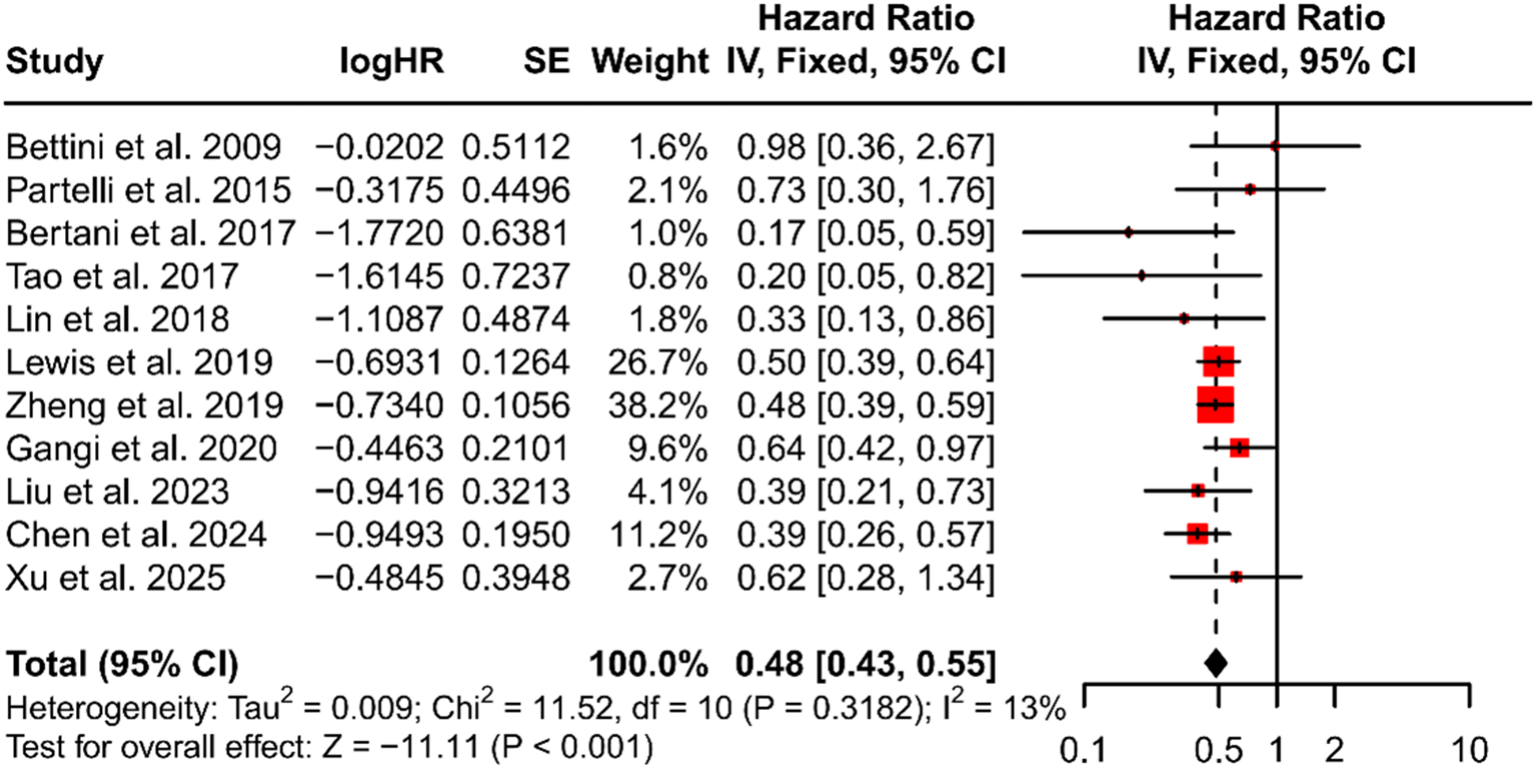
Forest plot that compare overall survival using the pooled HR. HR, hazard ratio; CI, confidence interval.

**Figure 3:**
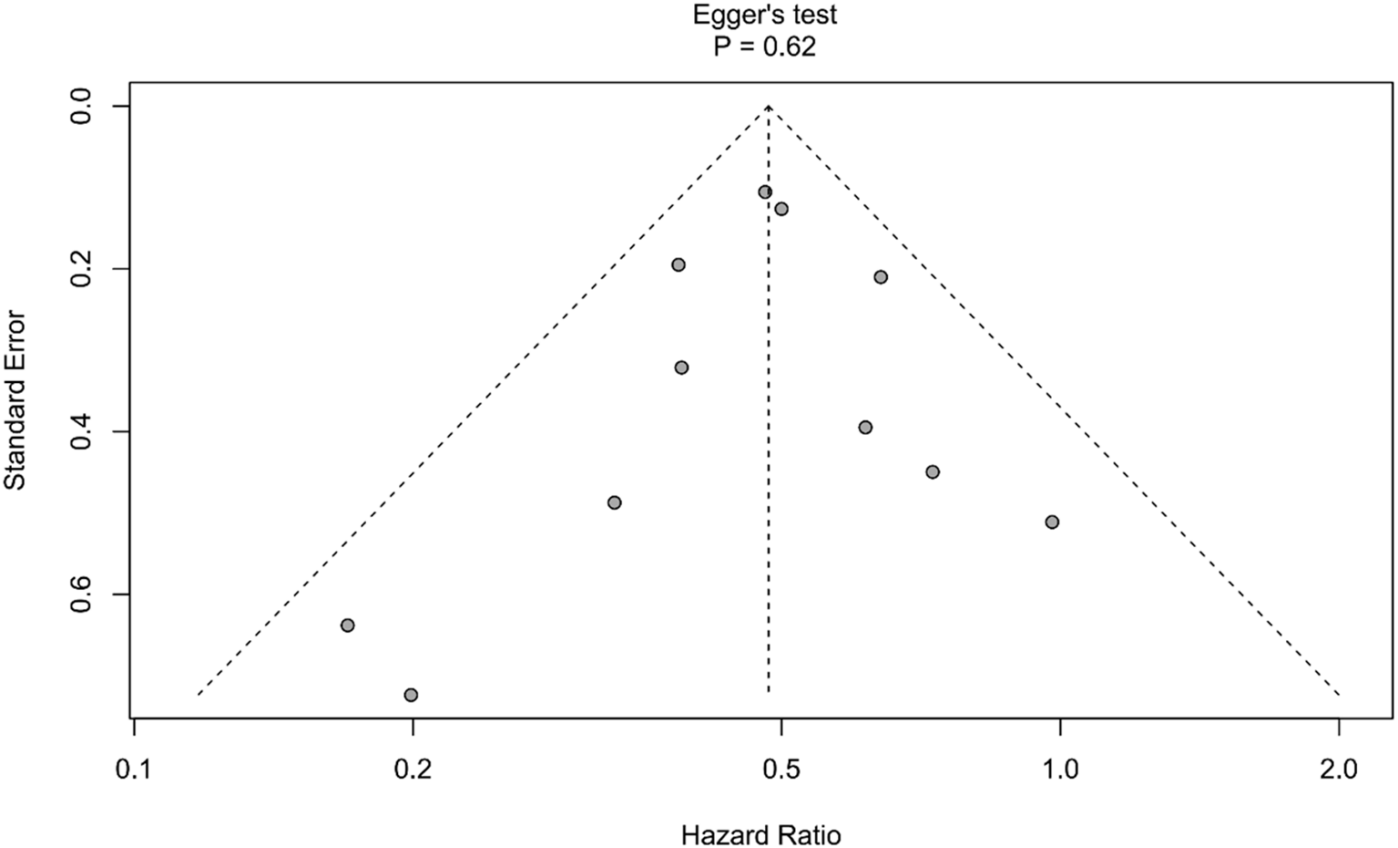
Funnel plot for assessing the risk of bias in studies.

**Figure 4:**
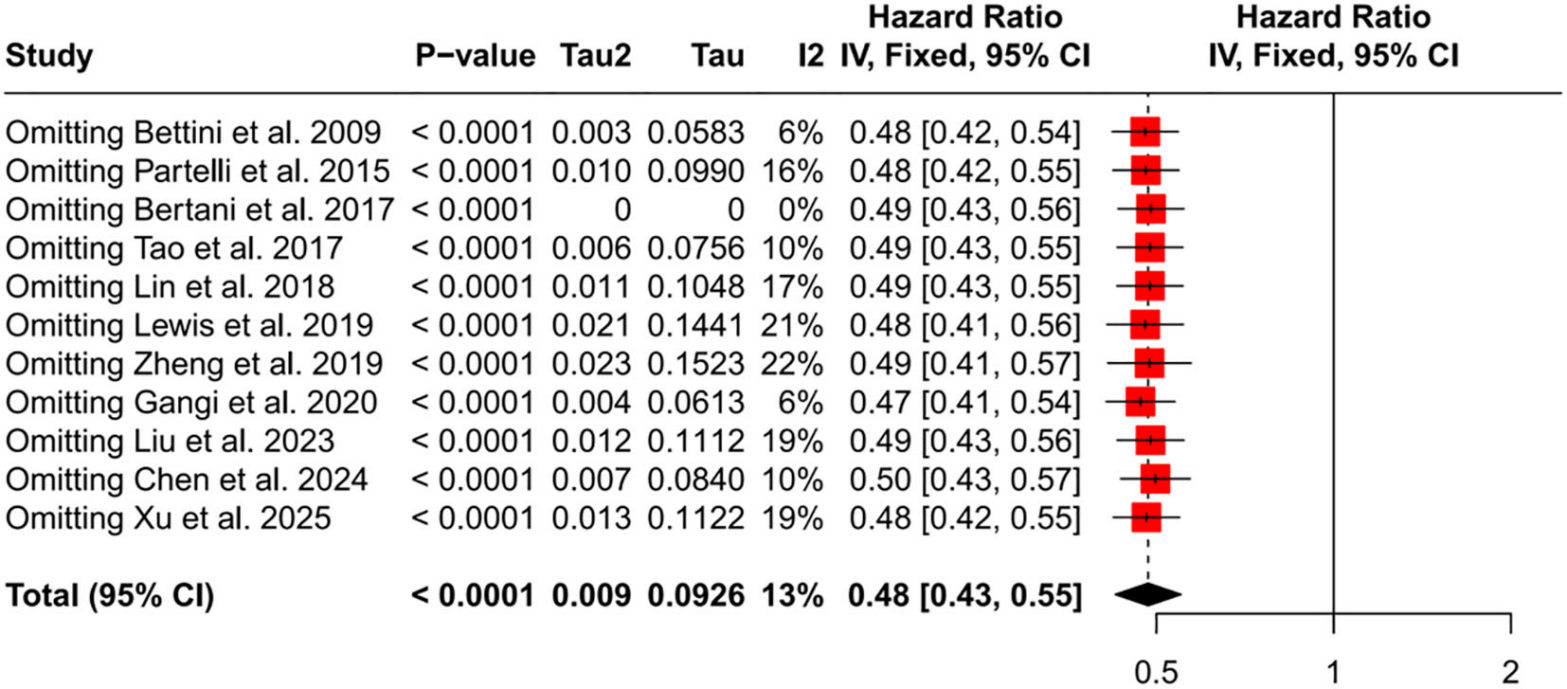
Forest plot of sensitivity analysis. HR, hazard ratio; CI, confidence interval.

All included studies used HR to compare OS between patients who underwent PTR and those who did not. The meta-analysis found a pooled HR of 0.48 for PTR recipients versus non-recipients (95% CI: 0.43–0.55, *P* < 0.001), indicating a significant OS advantage for patients treated with PTR (**Figure 2**). Between-study heterogeneity was low (*I^2^* = 13%), and there was no evidence of publication bias (Egger’s test *P* = 0.62, **Figure 3**). Sensitivity analysis further confirmed the robustness of the result: sequential removal of individual studies did not materially change the pooled estimate (**Figure 4**).

### 3.6 Subgroup analysis

**Figure 5:**
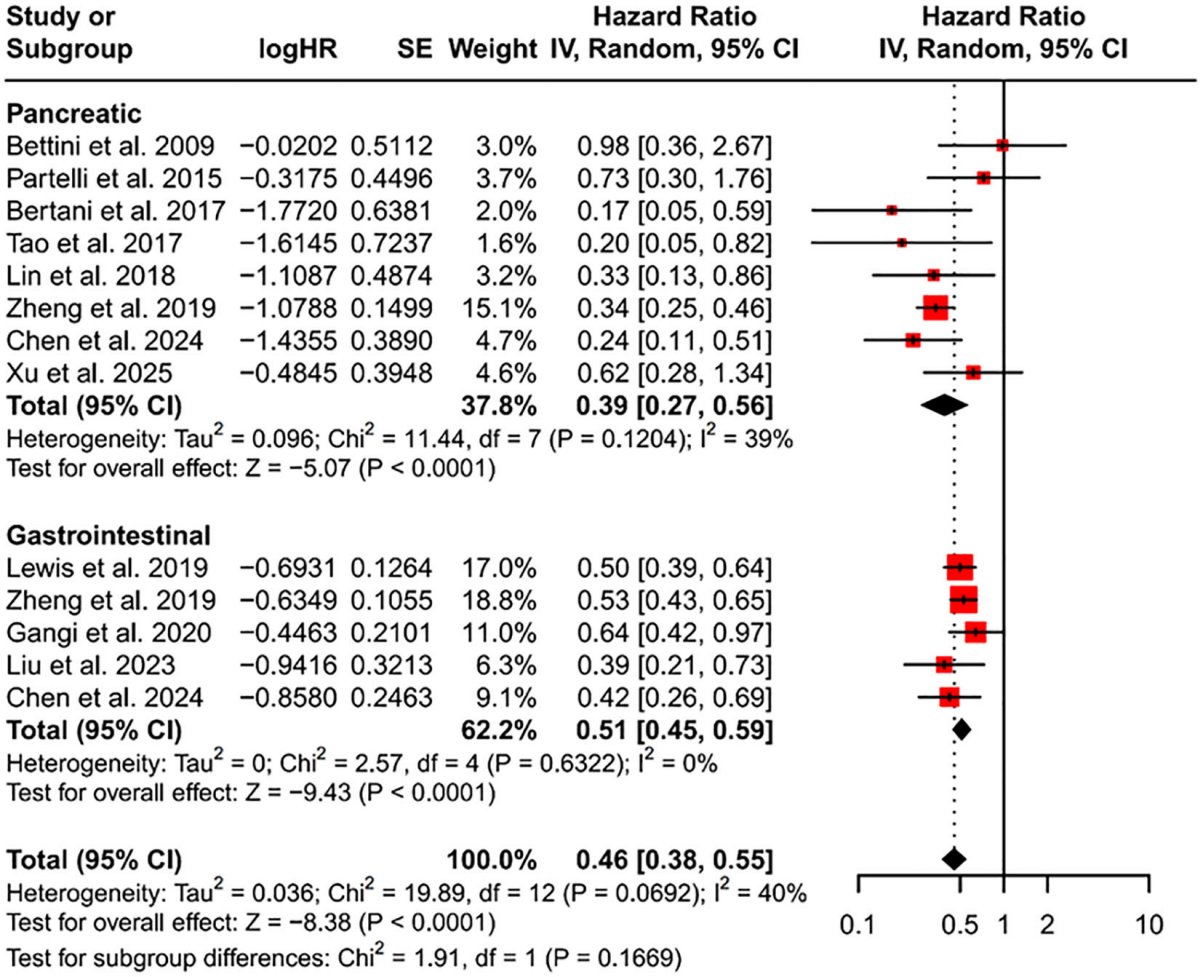
Forest plot of subgroups according to primary tumor location. HR, hazard ratio; CI, confidence interval.

We conducted a subgroup analysis by primary tumor site (pancreas versus gastrointestinal tract) (**Figure 5**). Because one subgroup showed *I^2^* = 39%, we applied a random-effects model. For primary pancreatic neuroendocrine tumors (P-NETs), the pooled HR was 0.39 (95% CI, 0.27–0.56). For primary GI-NETs, the pooled HR was 0.51 (95% CI, 0.45–0.59). Although the pooled HR for GI-NETs is numerically higher than that for P-NETs, the GI-NET subgroup exhibited virtually no heterogeneity (*I^2^* = 0%) and contributed a larger weight (62.2%).

**Figure 6:**
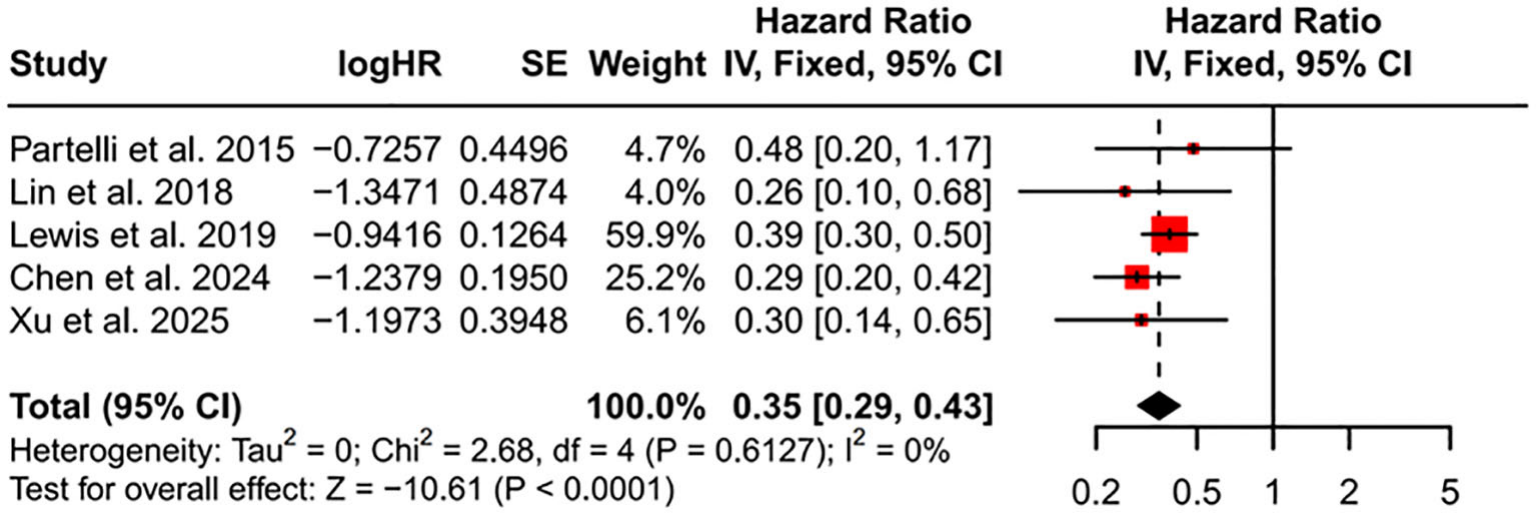
Forest plot of PTR combined with LR. HR, hazard ratio; CI, confidence interval; PTR, primary tumor resection; LR, liver resection.

Five studies (23, 25, 27–29) compared OS between patients who received PTR plus LR and those who did not undergo resection. These results were pooled in a meta-analysis (**Figure 6**), yielding a combined HR of 0.35 (95% CI, 0.29–0.43, *P* < 0.001). Thus, in patients with resectable LM, adding LR to PTR did not compromise the survival benefit associated with PTR.

## 4 Discussion

Due to the nonspecific nature of clinical symptoms, most patients with GEP-NETs are diagnosed at a late stage, often with distant metastases. LM is more than ten times more likely than metastasis to other sites (30). This prevalence is likely linked to the liver’s anatomical features. The portal vein directly channels venous blood from the gastrointestinal tract to the liver, and the hepatic sinusoids exhibit low shear (31). These conditions facilitate tumor cell colonization in the liver, leading to implantation metastasis. Clinically, metastasis to other locations is typically seen as an advanced stage, and surgical intervention is generally discouraged (32). This perception complicates surgical decision-making for patients with GEP-NETs and LM. The role of surgery in patients with advanced GEP-NETs and distant metastases is debated. Some studies suggest that PTR can reduce tumor burden, potentially extending patient survival even with LM (8–10). Conversely, other research argues that PTR does not significantly improve OS compared to non-resection and may increase mortality risk due to surgical complications (13, 14). Given these clinical complexities, we conducted a meta-analysis to evaluate the value and role of PTR in the treatment strategy for GEP-NETs.

Previous meta-analysis have examined the efficacy of palliative resection for midgut neuroendocrine tumors with unresectable LM (33, 34). Our study, however, uniquely includes articles on radical resection. Palliative surgery, often performed in patients with unresectable LM, primarily aimed at alleviating symptoms, such as obstruction, bleeding, or hormonal imbalances, rather than providing a curative outcome. In contrast, radical surgery involved both PTR and LR and was typically performed in patients with technically resectable LM, aiming for curative intent. Our study attempts to address this gap by including both surgical approaches, offering a broader view of the potential benefits of surgery in patients with GEP-NETs with LM. Additionally, our study encompassed tumors located in the stomach and pancreas, further categorized by primary tumor location. By applying stringent exclusion criteria, we excluded low-quality studies and incorporated recent literature. As one of the earliest meta-analyses to investigate surgical treatment for patients with GEP-NETs with LM, our study demonstrated a significant survival benefit from PTR for these patients (HR = 0.48, 95% CI: 0.43-0.55, *P* < 0.001). When LM are resectable, combining LR may enhance this survival advantage, but it should be considered only for highly selected patients.

Retrospective studies and international guidelines suggest that patients eligible for surgical treatment typically have well-differentiated G1 or G2 tumors, good performance status, a limited and resectable LM burden, and no uncontrolled extrahepatic disease. Additionally, symptomatic primary tumors, such as those causing obstruction, bleeding, or hormone-related symptoms, often indicate the need for surgery. However, these clinicopathological characteristics were not consistently reported in the studies reviewed, complicating the assessment of whether the observed survival benefits are due to the surgery itself or selection bias. To better identify patient subgroups that may benefit most from surgical management, further prospective studies with standardized reporting on metastatic burden, tumor grade, surgical intent, and symptom status are necessary.

The survival benefit observed may result from several mechanisms. Firstly, resecting the primary tumor significantly reduces tumor burden and decreases the overall proliferative activity of tumor cells, thereby slowing the progression of LM (20). Secondly, a study by Bertani et al. (35) suggests that PTR enhances the efficacy of PRRT for P-NETs with LM, extending median OS from 65 to 112 months (*P* = 0.011). Additionally, PTR can eliminate the source of LM, alleviate symptoms like “carcinoid” syndrome, and improve systemic conditions. For instance, surgery-induced reductions in serotonin levels in patients with “carcinoid” syndrome can mitigate organ damage caused by symptoms such as diarrhea and flushing (36). Lastly, the continued growth of the primary lesion may lead to severe complications, including intestinal obstruction, gastrointestinal bleeding, or biliary obstruction. According to ESMO guidelines, PTR for small bowel neuroendocrine neoplasms reduces the risk of intestinal obstruction and improves patient prognosis (37). In cases of colorectal neuroendocrine neoplasms, palliative resection significantly lowers the risk of local infiltration and bleeding, even with liver metastasis, if the primary lesion is ≥ 4 cm in diameter, thereby prolonging survival (20).

Our findings underscore the long-term benefits of surgical treatment over conservative management, yet several critical issues warrant attention. Primarily, it is essential to identify which patients will benefit from surgery in terms of survival. Based on the reviewed articles, we have summarized several strategies for reference. Numerous studies (20, 23, 25, 27, 29) emphasize the importance of differentiating the primary tumor location. For instance, primary tumors in the pancreas often lead to severe postoperative complications, making resection generally inadvisable. However, in cases of distal pancreatectomy, the complications are typically manageable, making surgical intervention a viable option. Additionally, the decision to operate on high-grade tumors remains contentious. A recent consensus by the European Society of Endocrine Surgeons (ESES) advises cautious surgical exploration for patients with high-grade tumors (38). These perspectives may soon contribute to establishing a widely accepted standard for tumor resection.

This study has several limitations. First, it only included retrospective cohort studies, which inherently carry a high risk of bias (39, 40). The primary bias stems from missing critical information in most studies, such as survival analysis data for the surgical resection group, reasons for non-resection, and details on drug therapies. These gaps can significantly impact treatment decisions and lead to variations in survival outcomes. Additionally, since all included studies used non-randomized designs, selection bias is unavoidable (41). In clinical practice, surgical interventions are usually reserved for patients with better physical conditions, slower disease progression, and less aggressive tumors (42). Without proper statistical adjustments, selection bias can systematically skew study results. Although some studies attempted to mitigate this issue through statistical adjustments, the risk of bias persists, potentially undermining the research’s effectiveness (22, 23, 26). Thus, there is an urgent need for randomized controlled trials to further validate the role of surgical resection. Furthermore, only three studies in this meta-analysis (20, 22, 23) specifically reported OS results for patients with small intestinal NETs. Consequently, a more detailed subgroup analysis to differentiate the efficacy of PTR in P-NETs and small intestinal NETs is not feasible. As a result, the current subgroup findings primarily reflect data that combine small intestinal NETs with other GI-NETs, potentially obscuring site-specific differences.

Future studies should prioritize identifying the benefits and risks for patients undergoing PTR, as these insights are vital for surgeons to make informed clinical decisions. This is particularly important for elderly patients, who often have poor physical health and multiple chronic conditions. For these patients, determining whether surgical intervention will yield the desired clinical outcomes is a primary concern for surgeons when developing treatment plans. Conducting standardized studies to address these issues will ultimately result in more meaningful clinical outcomes.

## 5 Conclusion

In conclusion, patients with GEP-NETs and LM appear to derive a survival benefit from PTR compared with conventional therapy. However, clear selection criteria are needed to identify which patients will truly benefit from a surgical approach. In addition, synchronous LR in patients with resectable LM may further enhance this survival advantage.

## Data Availability

The original contributions presented in the study are included in the article/**supplementary material**. Further inquiries can be directed to the corresponding author.
