# Supplementary material for: Resection of the primary tumor with or without liver resection reduces the risk of death in patients with liver metastatic gastroenteropancreatic neuroendocrine tumors: a systematic review and meta-analysis
Source: Front Oncol. 2026 Jan 14;15:1693647. doi: 10.3389/fonc.2025.1693647 (PMC12846988; doi:10.3389/fonc.2025.1693647)
Supplement: Supplementary file 1 [file Table1.docx]

# Supplementary Table 1. PRISMA-S Checklist.

| **Item** | **Description** | **Details** |
| --- | --- | --- |
| S1 | Database(s) and other sources searched | PubMed, Embase |
| S2 | Platform/interfaces used | PubMed interface; Embase (Elsevier) |
| S3 | Study registries searched | Not applicable |
| S4 | Web resources / grey literature | Not applicable |
| S5 | Full search strategy for each database | See Supplementary Table 2: Full Search Strategy |
| S6 | Limits and filters applied | Date range May 15, 2005–May 15, 2025; Humans; English (if applied) |
| S7 | Search dates | May 15, 2025 |
| S8 | Years covered | May 15, 2005–May 15, 2025 |
| S9 | Controlled vocabulary use (MeSH/Emtree) | Not used; free-text terms only |
| S10 | Translation across databases | Identical free‑text terms used across databases |
| S11 | Citation management software | EndNote X9 |
| S12 | Deduplication process | Automatic + manual deduplication in EndNote X9 |
| S13 | Screening process/tools | Manual title/abstract and full‑text screening |
| S14 | Search updates | No additional updates beyond May 15, 2025 |
| S15 | PRISMA flow diagram | Provided in the manuscript |
| S16 | Full documentation of search strategy | Included in Supplementary Table 2 and this checklist |

# Supplementary Table 2. Full Search Strategy.

| ****Database**** | ****Search Strategy**** |
| --- | --- |
| PubMed | ("liver metastasis"[tiab] OR "liver metastases"[tiab]) AND ("neuroendocrine tumor"[tiab] OR NET[tiab]) AND (surgery[tiab] OR resection[tiab]) AND ("2005/05/15"[Date - Publication] : "2025/05/15"[Date - Publication]) |
| Embase | ('liver metastasis'/exp OR 'liver metastases':ti,ab OR 'liver metastatic':ti,ab) AND ('neuroendocrine tumor':ti,ab) AND ('surgery'/exp OR 'resection':ti,ab) AND [2005-05-15]/sd NOT [2025-05-15]/sd |

# Supplementary Table 3. PICOS criteria.

| P – Population | Patients with gastroenteropancreatic neuroendocrine tumors (GEP-NETs) accompanied by liver metastases (LM). |
| --- | --- |
| I – Intervention | Primary tumor resection (PTR), regardless of whether liver resection was performed. |
| C – Comparison | Non-resection group (patients who did not undergo primary tumor resection). |
| O – Outcomes | Overall survival (OS); studies must provide HR and 95% CI, either directly or extractable from survival curves. |
| S – Study Design | Comparative observational studies (retrospective or prospective cohort). Excluded: case reports, reviews, meeting abstracts, guidelines, non-English publications, and single-arm studies. When duplicate study populations existed, only the highest-quality study was included. |
